# Interpenetrating Polymer Networks as Innovative Drug Delivery Systems

**DOI:** 10.1155/2014/583612

**Published:** 2014-05-14

**Authors:** Alka Lohani, Garima Singh, Shiv Sankar Bhattacharya, Anurag Verma

**Affiliations:** School of Pharmaceutical Sciences, IFTM University, Moradabad, Uttar Pradesh 244102, India

## Abstract

Polymers have always been valuable excipients in conventional dosage forms, also have shown excellent performance into the parenteral arena, and are now capable of offering advanced and sophisticated functions such as controlled drug release and drug targeting. Advances in polymer science have led to the development of several novel drug delivery systems. Interpenetrating polymer networks (IPNs) have shown superior performances over the conventional individual polymers and, consequently, the ranges of applications have grown rapidly for such class of materials. The advanced properties of IPNs like swelling capacity, stability, biocompatibility, nontoxicity and biodegradability have attracted considerable attention in pharmaceutical field especially in delivering bioactive molecules to the target site. In the past few years various research reports on the IPN based delivery systems showed that these carriers have emerged as a novel carrier in controlled drug delivery. The present review encompasses IPNs, their types, method of synthesis, factors which affects the morphology of IPNs, extensively studied IPN based drug delivery systems, and some natural polymers widely used for IPNs.

## 1. Introduction

In the present scenario polymers are among the largest volume chemical products in the world and the global market for polymer products is growing rapidly. Polymers have always been valuable excipients in tablet and capsule formulations [1] and also have shown excellent performance into the parenteral arena as blood circulation time enhancers [2] and are now capable of offering advanced and sophisticated functions such as controlled drug release and drug targeting [3]. In the recent decades, an ever growing demand for improved polymer properties has paved the development of the blending of polymer mixtures [4, 5]. In order to overcome the poor biological performance and to improve mechanical strength a new class of polymers has been introduced which are based on blending of either natural or synthetic polymers alone or in combinations.

**Figure 1:**
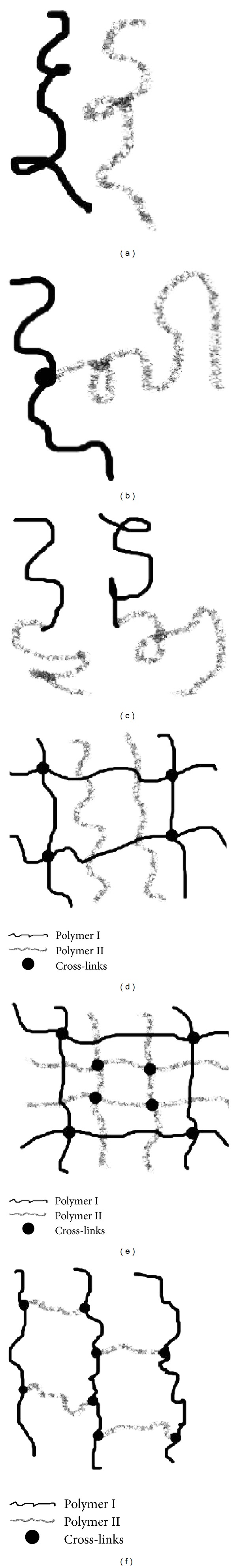
(a) A polymer blend; (b) a graft copolymer; (c) a block copolymer; (d) semi-IPN; (e) full IPN; F- cross-linked copolymer.

An interpenetrating polymer network (IPN) is defined as a blend of two or more polymers in a network with at least one of the systems synthesized in the presence of another [6]. This results in a formation of physically cross-linked network when polymer chains of the second system are entangled with or penetrate the network formed by the first polymer. Each individual network retains its individual properties so synergistic improvements in properties like strength or toughness can be seen [7]. An IPN can be distinguished from polymer blend in the way that an IPN swells but does not dissolve in solvents and creep and flow are suppressed [8]. They are also different from graft copolymers and polymer complex that involve either chemical bonds and/or low degree of cross-linking. From this point of view only, IPN can be generally named “polymer alloys” through which polymer blends can be made chemically compatible to achieve the desired phase morphology [9]. IPN can be distinguished from the other multiple systems through their bicontinuous structure ideally formed by cross-linking of two polymers that are in intimate contact but without any chemical contact and yields a material with improved properties depending on the composition and degree of cross-linking (Figure 1). The concept of IPN goes back at least as far as 1914 when the first interpenetrating polymer network was invented by Aylsworth [10]. This was a mixture of natural rubber, sulphur, and partly reacted phenol-formaldehyde resins. The term IPN was introduced for the first time by Miller in 1960s in a scientific study about polystyrene networks [11]. Since that time the field of IPN has expended dramatically.

Advances in polymer science have led to the development of several novel drug delivery systems. IPNs have shown superior performances over the conventional individual polymers and, consequently, the ranges of applications have grown rapidly for such class of materials. The advanced properties of IPNs have attracted considerable attention in pharmaceutical field especially in the area of drug delivery. These biocompatible, nontoxic, and biodegradable polymer networks are now acquiring unique place in delivering bioactive molecules, particularly in controlled and targeted drug delivery applications. Various research investigations have shown that a variety of drugs can be delivered effectively via IPN based delivery systems (Table 1).

## 2. Classification of IPN 

### 2.1. Based on Chemical Bonding [12]

#### 2.1.1. Covalent Semi-IPN

When two separate polymer systems that are cross-linked form a single polymer network, it is called covalent semi-IPN.

#### 2.1.2. Noncovalent Semi-IPN

In noncovalent semi-IPNs only one of the polymer systems is cross-linked.

#### 2.1.3. Noncovalent Full IPN

A noncovalent full IPN is one in which the two separate polymers are cross-linked independently.

### 2.2. Based on Method of Synthesis

IPNs are of different types: sequential IPN, subsequent IPN, latex IPN, gradient IPN, and thermoplastic IPN.

#### 2.2.1. Sequential IPN

**Figure 2:**
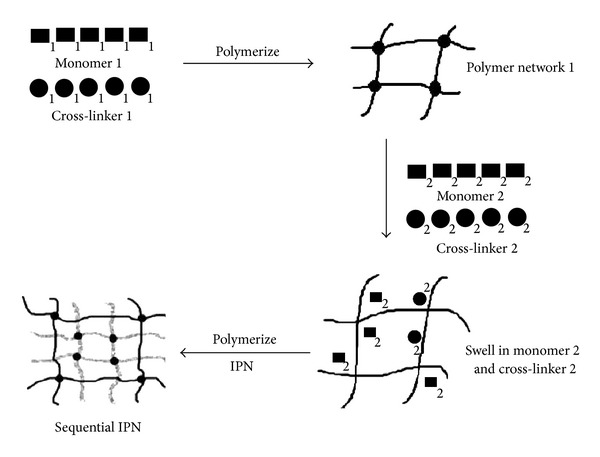
Sequential IPN formation.

In sequential IPN, the first cross-linked polymer network is swollen by the monomer of the second polymer that is polymerized and/or cross-linked afterwards. In this class an IPN is formed by polymerizing the first mixture of monomer (I), cross-linker, and initiator to form a network. The network is swollen with the second combination of monomer (II) and cross-linker which is polymerized to form an IPN [6]. Sequential IPNs are easy to synthesize. The primary requirement is that monomer (II) and coreactants swell properly into polymer network I. Usually elastomers are used for network I because they swell easily compared to glassy network (Figure 2).

#### 2.2.2. Simultaneous IPN

**Figure 3:**
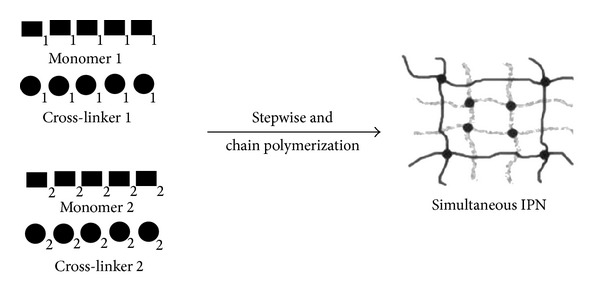
Formation of simultaneous IPN.

An IPN is formed by polymerization of two different monomer and cross-linking agent pairs together in one step [6, 7]. The key to the success of this process is that the two components must polymerize simultaneously by noninterfering routes (Figure 3).

#### 2.2.3. Latex IPN

The common problem associated with most IPNs is the difficulty in molding after they are formed since they are thermosets. One way to overcome this problem is to use latex IPN. They are also called interpenetrating elastomeric networks especially when both polymers are above the glass transition temperature. In latex type IPN both networks are included in a single latex particle, usually by polymerization of the second monomer together with the cross-linking agent and activator in the original seed latex of the first cross-linked monomer [6]. Latex IPNs are formed from a mixture of two lattices, frequently exhibiting a “core” and “shell” structure. In a sequential method, if the monomers corresponding to the second polymer react near the surface of the first polymer, latex IPN with shell/core morphology will be obtained [13].

#### 2.2.4. Thermoplastic IPN

These IPNs have completely erased the idea of chemical cross-linkers and use physical cross-linkers, like thermoplastic elastomers. The thermoplastic IPNs are combination of two physically cross-linked polymers [6, 7]. Typical physical cross-links arise from ionic groups, crystallinity, or glassy domains. Thus, these materials flow at elevated temperatures, similar to the thermoplastic elastomers, while behaving like conventional thermoset IPNs and at their application temperature usually at least one component is a block copolymer and the other one a semicrystalline or glassy polymer [9]. Depending on the continuity and proportion of phases, this kind of IPNs can exhibit a wide range of properties, from reinforced rubber to high impact plastics.

#### 2.2.5. Gradient IPN

Gradient IPNs have compositions which vary as a function of position in the sample. They are formed as a result of the swelling of the first monomer network in the network of the second monomer. Before equilibrium is established a stage comes where swelling is terminated and polymerization is carried out to produce the IPN. In this type of system the concentration of second monomer network has a gradient over the first monomer network [6, 7, 30].

## 3. Preparation of IPN

### 3.1. Casting Evaporation

This method has been used widely to form cross-linked polymer network. In this method each polymer constituent is heated until it is dissolved and then added to cross-linker solution [31]. In case of sequential process, solution of polymer I is added to the cross-linker solution followed by addition of polymer II solution. In both cases the solution is heated and mixed and then casted and dried. IPN gels can be prepared by this technique.

### 3.2. Emulsification Cross-Linking

This method is based on phase separation. Generally single emulsion cross-linking technique is based on w/o emulsion but recently w/w emulsion method has also been developed to form IPN [32]. The main advantage of w/w emulsion method is that there is no use of organic solvents which might leave toxic residue that is incompatible with IPN biomaterials.

In w/o emulsification method the water soluble materials are dissolved in aqueous phase at specific temperature to form homogenous solution by stirring. This aqueous phase is added to oil phase to prepare w/o emulsion [33] but in w/w emulsion technique an aqueous solution of water soluble polymers is emulsified as a dispersed phase in an aqueous solution of another polymer that acts as continuous phase. Then the dispersed polymer phase is cross-linked to form IPN network [32].

### 3.3. Miniemulsion/Inverse Miniemulsion Technique

**Figure 4:**
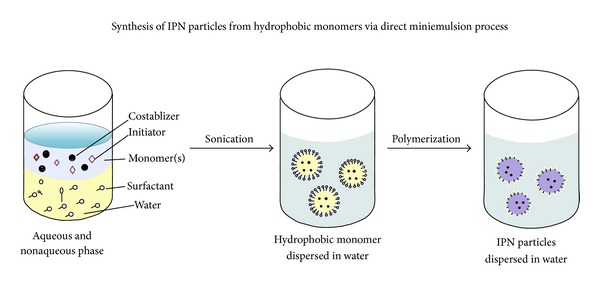
Synthesis of IPN particles by miniemulsion polymerization.

This technique allows one to create small stable droplets in a continuous phase by the application of high shear stress [34]. The idea of miniemulsion polymerization is to initiate the polymer in each of the small stabilized droplets. To prevent the degradation of miniemulsion through coalescence, a surfactant and a costablizer are added that are soluble in dispersed phase but insoluble in continuous phase. This process of IPN formation can be divided into three steps. In the first step, constituent polymers are obtained by sonication using specific initiator. In the second step, one of the constituent polymers is polymerized and cross-linked using a cross-linking agent. As a result a semi-IPN is formed till the second stage. In the third step, a full IPN is formed polymerizing and cross-linking the second constituent polymer by the addition of second cross-linker.  Figure 4 represents the formation of IPN particles by the process of direct (oil in water) miniemulsion polymerization.

In case of inverse miniemulsion (water in oil), hydrophilic monomers can be easily polymerized. In this case the monomer solution is miniemulsified in a continuous hydrophobic phase. The polymerization process can be initiated either from the continuous phase or from the droplet. Koul et al. synthesized novel IPN nanogels composed of poly(acrylic acid) and gelatin by inverse miniemulsion technique. Acrylic acid monomer stabilized around the gelatin macromolecules in each droplet was polymerized using ammonium persulfate and tetramethyl ethylene diamine and cross-linked with N, N-methylene bisacrylamide (BIS) to form semi-IPN nanogels, which were sequentially cross-linked using glutaraldehyde to form IPNs [35].

## 4. Factors That Affect IPN Morphology

Most IPN materials that have been investigated show phase separation. The phase however varies in amount, size, shape, and sharpness of their interfaces and degree of continuity. These aspects together constitute the morphology of IPN which includes chemical compatibility of the polymers, interfacial tension, cross-linking densities of the networks, polymerization methods, and IPN composition. Compatibility between polymers is necessary for IPNs because monomers or prepolymers must be in solution or swollen networks during synthesis. Phase separation generally ensues in the course of polymerization, but the resulting phase domain size is smaller for higher compatibility systems [13]. Increase in cross-linking density of polymer network I in an IPN clearly decreases the domain size of polymer II. This effect is reasonable because the tighter initial network must restrict the size of the regions in which polymer II can phase-separate. Polymer composition also plays an important role in IPN morphology. The IPN composition determines the relative amounts of the two phases present after polymerization. Increase in the amount of polymer II generally leads to increase in domain size, but effect depends upon method of polymerization [7]. Ratio of viscosity between dispersed phase and the viscosity of matrix also plays an important role in affecting the morphology of IPN. If the minor component of the blend has lower viscosity than a major component, that component will be homogenously dispersed. On the other hand the minor component will be coarsely dispersed if it has higher viscosity than the major component.

## 5. Typical Pharmaceutical Factors

### 5.1. Mechanical Strength

Mechanical property of IPN is very important for pharmaceutical applications. The integrity of the drug delivery system during the lifetime of the application is very important, unless the device is designed as a biodegradable system. A drug delivery system designed to protect a sensitive therapeutic agent must maintain its integrity to be able to protect the therapeutic agent until it is released out of the system. Changing the degree of cross-linking has been utilized to achieve the desired mechanical property of the IPN. Increasing the degree of cross-linking of the system will result in a stronger polymer network [36]. However, a higher degree of cross-linking can create a more brittle structure. Hence, there is an optimum degree of cross-linking to achieve a relatively strong IPN based drug delivery system. Copolymerization has also been utilized to achieve the desired mechanical properties [37].

### 5.2. Sterilization

Research in the field of IPNs for drug delivery applications has been intense in the last decade. IPN represents a new class of materials with better thermal stability than the individual polymer and it is very important during the processing of the materials and heat sterilization. Sterilization capability is a necessary requirement for these materials to be used in drug delivery or medical application. IPN based devices (such as drug delivery vehicles, implants, etc.) must be prepared under good manufacturing practice conditions and sterilized or disinfected (depending upon necessity) before medical use. The sterilization method (wet or dry heat, chemical treatment or radiation) should not cause structural changes or lead to chain scission, cross-linking, or a significant alteration in mechanical properties of IPN [38]. The traditional methods of sterilization include the use of dry or moist heat, chemicals (ethylene oxide), or radiation. Steam sterilization by autoclaving at 121°C is the most widely employed method but might induce melting and/or hydrolysis of the polymer matrix, which limits its use for most of the polymeric materials [39]. Chemical sterilization with ethylene oxide gas offers the advantage of effective treatment at ambient temperature and is useful for hydrolytically unstable polymers. Nevertheless, its popularity is decreasing due to the well-known toxicity and flammability of ethylene oxide.

High-energy radiation sterilization method has the advantage of high efficiency, negligible thermal effects. Polymers exhibiting high heats of polymerization tend to cross-link upon radiation, indicating an apparent increase in mechanical stability with increasing radiation doses [39, 40]. Radiation cross-linking does not involve the use of chemical additives and therefore retaining the biocompatibility of the biopolymer. Also, the modification and sterilization can be achieved in a single step and hence it is a cost-effective process to modify biopolymers having their end use specifically in biomedical application.

### 5.3. Large Scale Production

The major challenge of research and development of IPNs for drug delivery is large scale production. There is always a need to scale up laboratory or pilot technologies for eventual commercialization. It is easier to modify IPNs at laboratory scale for improved performance than at large scale. Maintaining concentration and composition of polymers at large scale is also a challenge. Despite the number of researches and patents for IPN drug delivery technologies, commercialization is still at its early stage. This is partially due to the fact that most of the research studies are carried out by researchers in academia. Nevertheless, greater effort is needed to bring IPN based drug delivery systems from the experimental level to the pilot scale production and extend their practical applications. This can be achieved by addressing several aspects, which include boosting the selectivity without compromising biocompatibility and stability, optimizing polymer modification techniques, using the proper engineering configurations, understanding the mechanism of transport, and using cost-effective materials and methods.

## 6. Preclinical Studies with IPNs

A preclinical study is a stage of research that begins before clinical trials (testing in humans) and during which important feasibility, iterative testing, and drug safety data is collected. The main goals of preclinical studies are to determine a product's ultimate safety profile. IPNs based on poly(acrylic acid) and gelatin were evaluated for in vivo biodegradation and release of gentamicin sulphate by Changez et al. [41] In vivo degradation studies demonstrated that the degradation and drug release depend on the composition of hydrogels. It was observed that the rate of in vivo degradation of hydrogels was much lower than in vitro degradation. In vivo drug release profile showed a burst effect, followed by controlled release. Drug concentration was measured in the local skin tissue, blood serum, kidney, liver, and spleen of male Wistar rats. The concentration of drug in local skin tissue was found to be higher than the minimum bactericidal concentration for a study time of 60 days. It was concluded that these delivery systems may have a good therapeutic potential for the treatment of localized infection like osteomyelitis. In another study Changez et al. evaluated the in vivo safety and efficacy of gentamycin sulphate (GS) or vancomycin hydrochloride (VCl) loaded IPN device [42]. The placebo and drug-loaded device (acrylic acid: gelatin: 1 : 1 w/w) were employed for the treatment of experimental osteomyelitis in rabbit. Rabbits were categorized into four groups and were treated with IPN device loaded with varying drug concentrations. After implantation of IPN device in the adjacent tissue of femoral cavity and serum the drug concentration was measured. On the 7th day maximum drug concentration was found in femoral cavity with all the devices. No drug was found after 21 days at the local site with devices containing 12 ± 1 mg of 22% w/w GS and 44% w/w GS whereas with 16 ± 1 mg device (44% w/w GS or VCl) drug was detected even after 6 weeks. Macroscopic evaluation after treatment revealed that swelling, redness, local warmth, and drainage decreased depending upon the drug loading of the implants. Sequential radiographs, histology, microbiologic assay, and scanning electron micrograph demonstrated that devices containing 16 ± 1 mg of 44% w/w GS or 44% w/w GS VCl are the most suitable devices, which heal the infection after 6 weeks of treatment. None of the IPN devices showed toxic level of drug in serum at any given time.

Kulkarni et al. synthesized pH responsive IPN hydrogel beads of polyacrylamide grafted *κ*-carrageenan and sodium alginate for targeting ketoprofen to the intestine and studied their in vivo performance for the release of drug to the target site (intestine) [43]. Stomach histopathology of albino rats indicated that the prepared IPN beads were able to retard the drug release in stomach leading to the reduced ulceration, hemorrhage, and erosion of gastric mucosa without any toxicity.

## 7. IPN Based Drug Delivery Systems

Development of suitable carrier systems for delivery of active pharmaceuticals always remains a major challenge. New technological advances have brought many innovative drug delivery systems. A variety of approaches have been investigated for the controlled release of drugs and their targeting to selective sites including hydrogel, microspheres, nanoparticles, tablet, capsule, and films. Some widely studied IPN based drug delivery systems are discussed here.

### 7.1. Hydrogel

In recent decades hydrogels have been extensively used as a smart biomaterial in many biomedical applications such as drug delivery and tissue engineering because of their excellent physical and chemical properties. Hydrogels are hydrophilic three-dimensional polymeric networks that are chemically cross-linked or physically entangled having excellent swelling capacity [44]. To improve the properties of the hydrogel networks, a new class of hydrogels based on the interpenetration of two-polymer network has been recently developed. IPN hydrogels are a subset of the general class of IPNs. IPN hydrogel offers a variety in the formulation of physical forms including microspheres, nanoparticles, and films. One of the most outstanding achievements of IPN hydrogels in drug delivery is the development of smart drug delivery systems (SDDS), also called stimuli-sensitive delivery systems. The concept of SDDS is based on conversation of physicochemical property of polymer systems upon an environmental stimulus, which includes physical (temperature, electricity, light, and mechanical stress) chemical (pH, ionic strength), or biological (enzymes) signals. Such stimuli can be either internal signals (as a result of changes in the physiological condition of a living subject) or external signals (artificially induced). This sensitive behavior of hydrogels has sparked particular interest in their use as drug delivery vehicle capable of controlling drug release and drug targeting. In a study pH-sensitive IPN hydrogel beads of ibuprofen were formulated to minimize the release of drug in acidic medium and to control the drug release in alkaline medium (phosphate buffer) depending on the need [45]. IPN beads were prepared by ionotropic gelation process using AlCl3 as a cross-linking agent. It was observed that the drug release in acidic medium (pH 1.2) was considerably slow and complete drug release was achieved in phosphate buffer (pH 6.8) within 210 to 330 min depending upon the formulation variables. It can be concluded that pH-sensitive IPN hydrogels could be used to target the drug in the desired region. Pescosolido et al. reported a novel class of hydrogels based on the interpenetration of two polysaccharide networks. In situ forming IPN hydrogels of calcium alginate and dextran hydroxyethyl-methacrylate were developed and evaluated for protein release as well as for the behavior of embedded cells. It was observed that after an initial burst release bovine serum albumin was gradually released from the IPN hydrogels for up to 15 days. Encapsulation of expanded chondrocytes in the IPNs revealed that cells remained viable and were able to redifferentiate. IPN was described as a promising system as injectable in situ forming hydrogels for protein delivery and tissue engineering applications [46].

### 7.2. Microspheres

Microspheres are another promising class of IPN based drug delivery systems. Microspheres are free flowing small spherical powder particles made up of natural or synthetic polymers having diameter in the range of 1 *μ*m to 1000 *μ*m [47]. IPN microspheres are considered a versatile carrier for controlled release and drug targeting applications due to their capability to encapsulate a wide variety of drugs, protection of drugs, increased bioavailability, biocompatibility, patience compliance, biodegradability, and sustained release characteristics. Xanthan gum facilitated superabsorbent polymeric microspheres by w/o emulsion cross-linking method which was successfully prepared and evaluated for sustained release of ciprofloxacin hydrochloride. IPN formation was confirmed by Fourier transform infrared spectroscopy (FTIR) and X-ray diffraction (XRD) analysis. Differential scanning calorimetry (DSC) study was performed to understand the dispersion nature of drug after encapsulation. In vitro drug release study was extensively evaluated to observe the sustained drug delivery from IPN microspheres [48]. In another study an anticancer drug (5 fluorouracil) was successfully encapsulated in carbohydrate grafted IPN microspheres to increase the bioavailability. The resulting IPN microspheres were found to have an ability to release the drug for more than 12 hours [49].

### 7.3. Nanoparticles

Polymer based nanoparticles have been developed since the early 1980s, when progress in polymer chemistry allowed the design of biodegradable and biocompatible materials for targeting the drug into the desired site [50]. Nanoparticles as a carrier in drug delivery have attracted much attention in the last few years and have undergone the most investigation in recent years for biomedical applications due to their wide range of applications including their size, surface area, magnetic and optical properties, and biological transport that are brought into the perspective of drug delivery. Recently, there has been increased interest in IPN nanoparticles for utilization as the smart drug delivery system in the field of controlled drug release, to meet the demand for better control of drug administration. The idea of IPN nanoparticles as drug carriers may be employed to modify or to control the drug distribution at the tissue, cellular, or subcellular levels. IPN nanoparticles can be either nanospheres or nanocapsules depending on the method of preparation. Nanospheres are polymeric matrix systems in which the drug is dispersed within the polymer throughout the particle. On the contrary, nanocapsules are vesicular systems, which are formed by a drug-containing liquid core (aqueous or lipophilic) surrounded by polymeric; thus nanocapsules may be considered a reservoir system.

Nanocomposites having antibacterial activity towards* Escherichia coli* were developed by Krishna Rao et al. The chitosan particles were prepared by desolvation followed by cross-linking with poly(ethylene glycol-dialdehyde), which was prepared with poly(ethylene glycol) in the presence of a silver nitrate solution. The developed nanocomposites were characterized using UV-visible, FTIR, XRD, SEM, and TEM to understand their physicochemical properties. It was observed that prepared nanocomposites showed good antibacterial activity towards* E. coli* [51]. In a recent study it was observed that the novel IPN nanoparticles can be used to form in situ thermogelling devices for controlled protein release. IPN nanoparticles were synthesized by using poly(oligo(ethylene glycol) methyl ether methacrylate-co-oligo(ethylene glycol) ethyl ether methacrylate)-poly(acrylic acid). Atomic force microscopic images confirmed the homogenous and monodisperse morphology of the IPN nanoparticles. The study demonstrated that the IPN nanoparticles exhibit thermogelling properties at body temperature and these IPN nanoparticles allow their ease of injection and then slow release of protein. Histological analysis showed that following subcutaneous implantation IPN implants exerted minimal inflammation [52].

### 7.4. Tablets

Mandal et al. developed calcium ion cross-linked IPN matrix tablets of polyacrylamide-grafted-sodium alginate and sodium alginate for sustained release of diltiazem hydrochloride. Formulation of IPN structure was confirmed using FTIR spectroscopy. It was found that the relative magnitude of swelling capacity of IPN matrix and viscosity of the gel formed following dissolution of the polymers governs the drug release from IPN matrix [53]. The application of IPN tablets for controlled release of a water soluble antihypertensive drug (propranolol hydrochloride) was investigated by Kulkarni et al. The IPN tablets were prepared by a wet granulation/covalent cross-linking method. FTIR confirmed the cross-linking reaction and IPN formation, while XRD and SEM studies confirmed the amorphous dispersion of the drug within the IPN tablets. It was observed that the plain drug was released within 1 hr, while drug release from the resinate was prolonged for 2.5 hrs and the IPN matrices showed drug release up to 24 hrs [54].

## 8. Some Natural Polymers Used for IPN

### 8.1. Chitin

Chitin is the natural polysaccharide composed of *β* (1→4)-linked 2-acetamido-2-deoxy-*β*-D-glucose (N-acetylglucosamine) [55]. The principle derivative of chitin is chitosan. Chitin and chitosan are naturally available polymers having excellent properties such as biodegradability, biocompatibility, nontoxicity, and adsorption. Chitosan based IPN systems have gained considerable attention as a vehicle for drug delivery systems. A number of chitosan based IPN delivery systems have been developed to explore the useful features of chitosan.

Rani et al. reported use of pH-sensitive IPN beads composed of chitosan-glycine-glutamic acid cross-linked with glutaraldehyde for controlled drug release. It was observed that the swelling behavior and release of drug depend on pH, degree of cross-linking, and their composition. The results of this study indicated that the IPN beads of chitosan-glycine-glutamic acid might be useful for controlled release of drug [56]. In a study Reddy et al. synthesized ghatti gum and chitosan IPN microparticles by emulsion-cross-linking method using glutaraldehyde as a cross-linker. IPN microparticles were used to deliver diclofenac sodium to the intestine. Characterization of IPN microparticles was done by SEM, DSC, and FTIR and was evaluated for in vitro drug release. FTIR studies assessed the formation of IPN structure. It was found that the drug release from IPN microparticles was extended up to 12 hrs [57].

### 8.2. Carrageenan

Carrageenan is a high molecular weight linear polysaccharide comprising repeating galactose units and 3,6-anhydrogalactose (3,6 AG), both sulfated and nonsulfated, joined by alternating *α*-(1,3) and *β*-(1,4) glycosidic links [58]. It is obtained from edible red seaweeds. The name carrageenan has been originated from the* Chondrus crispus *species of seaweed known as carrageen moss or Irish moss. There are three basic types of carrageenan—kappa (*κ*), iota (*ι*), and lambda (*λ*). The *λ*-type carrageenan results in viscous solutions but is nongelling, while the *κ*-type carrageenan forms a brittle gel. The *ι*-type carrageenan produces elastic gels [59]. Mohamadnia et al. developed pH-sensitive IPN hydrogel beads of carrageenan-alginate for controlled drug delivery of betamethasone acetate. The effect of temperature and pH of the preparative media on the drug loading efficiency was investigated. Maximum loading efficiency was observed at pH 4.8 and 55°C. The chemical structure and morphology of the carrageenan IPN hydrogel with and without drug was studied using FTIR and SEM analyses [60].

### 8.3. Alginates

Alginic acid also called algin or alginate is an anionic polysaccharide found in brown seaweed and marine algae such as* Laminaria hyperborea, Ascophyllum nodosum, *and* Macrocystis pyrifera *[61]. Alginic acid is converted to its salt like sodium alginate and potassium alginate. Sodium alginate has been studied a lot for IPN drug delivery systems. Swamy et al. studied on thermoresponsive sodium alginate-g-poly(vinyl carpolactam) IPN beads as drug delivery matrices of an anticancer of an anticancer drug. They reported that the thermoresponsive IPN beads had higher drug release at 25°C than that at 37°C [62]. In a study pH-sensitive IPN hydrogel composed of a water soluble chitosan derivative (*N*,*O*-carboxymethyl chitosan) and alginate was synthesized by Chen et al. for controlling protein drug delivery. Genipin was used as a cross-linker to form a semi-interpenetrating polymeric network within the developed hydrogel system. The results clearly suggested that the synthesized IPN hydrogel could be a suitable polymeric carrier for site-specific protein drug delivery in the intestine [63].

### 8.4. Xanthum Gum

Xanthan gum is a high molecular weight, anionic extracellular polysaccharide that is produced by the gram-negative bacterium* Xanthomonas campestris*. It is widely used in food, cosmetics, and pharmaceuticals because of its encouraging reports on safety. In pharmaceuticals xanthum gum is used as thickening, suspending, and emulsifying agent [64, 65]. Bhattacharya et al. synthesized IPN hydrogel microspheres of xanthan gum based superabsorbent polymer and poly(vinyl alcohol) by water-in-oil emulsion cross-linking method for sustained release of ciprofloxacin hydrochloride. IPN formation was confirmed by FTIR and XRD analysis. It was reported that drug-loaded IPN microspheres were suitable for sustained drug release application [32].

### 8.5. Guar Gum

Guar gum is the powder of the endosperm of the seeds of* Cyamopsis tetragonolobus *Linn. (Leguminosae) [66]. Guar gum has recently been reported as an inexpensive and flexible carrier for oral extended release drug delivery [67]. In pharmaceuticals, it is used as tablet binder, suspending, disintegranting, stabilizing, and thickening agent and also as a controlled release drug carrier. Reddy et al. reported chitosan-guar gum based semi-IPN microspheres for controlled release of cefadroxil. Drug was loaded into the microspheres and cross-linked with glutaraldehyde, leading to the formation of a semi-IPN structure. XRD and DSC studies indicated that drug is dispersed at the molecular level in the semi-IPN matrix. It was reported that the drug was released from semi-IPN microspheres in a sustained and controlled manner for up to 10 hrs [68].

### 8.6. Locust Bean Gum

Locust bean gum is a branched, high molecular weight polysaccharide and is extracted from the seeds of carob tree* Ceratonia siliqua*. It consists of a (1, 4)-linked *β*-D-mannopyranose with branch points from their 6-positions linked to *α*-D-galactose (1,6-linked *α*-D-galactopyranose) [69]. Kaity et al. developed novel IPN microspheres of locust bean gum and poly(vinyl alcohol) for oral controlled release of buflomedil hydrochloride. It was reported that the microspheres showed control drug release property without any sign of incompatibility in IPN device [15]. Dey et al. developed IPN network of etherified locust bean gum and sodium alginate through ionotropic gelation with Al^3+^ ions and the drug release was compared with homopolymer networks. The degree of reticulation in IPNs was explained by the tensile strength measurement, neutralization equivalent, and drying kinetics of drug-free hydrogels. It was reported that IPNs had better mechanical strength than homopolymer network and also IPNs afforded maximum drug entrapment efficiency and showed drug release profiles up to 8 hours [70].

## 9. Conclusion

IPN represents very important field in drug delivery, which has various advantages like excellent swelling capacity, specificity, and mechanical strength which play an important role in controlled and targeted drug delivery. By developing IPN system using various polymers one has the opportunity of obtaining materials with a range of properties that will improve the properties and will overcome the disadvantages of individual polymer network.

## Figures and Tables

**Table 1:** Delivery of variety of drugs via different IPN based novel carriers.

Drug	Therapeutic category	Carrier system	Inference	Reference
5-Fluorouracil and diclofenac sodium	Antimetabolite and anti-inflammatory	Hydrogels	Localized drug delivery	[14]
Buflomedil hydrochloride		Microspheres	Controlled oral delivery of highly water soluble drug	[15]
5-Fluorouracil	Antimetabolite	Hydrogels	pH sensitivity to localize drug delivery	[16]
Diclofenac sodium	Anti-inflammatory	Microbead	Intestine specific drug release with minimum gastric side effects of drug	[17]
Aspirin	Anti-inflammatory	Microparticles	Intestinal specific drug delivery	[18]
Chlorpheniramine maleate	Antihistaminics	Beads	Controlled drug delivery	[19]
Ofloxacin hydrochloride	Antibacterial	Beads	Sustained drug release	[20]
Simvastatin	Hypolipidemic	IPN beads	Controlled drug release	[21]
Clarithromycin	Anti-*Helicobacter pylori *	Hydrogels	Stomach-specific drug delivery	[22]
Cloxacillin	Antibiotic	Floating beads	Prolonged sustained release in simulated gastric fluid	[23]
Bovine serum albumin	Serum albumin	Hydrogel beads	Improved drug release in physiological saline solution	[24]
Insulin	Hypoglycaemic	Microparticles	Innovative carrier for oral insulin delivery	[25]
Cefadroxil	Antimicrobial	Microgels	pH dependent extended drug release	[26]
Metformin hydrochloride	Hypoglycaemic	Mucoadhesive microspheres	Prolonged gastric residence with sustained release	[27]
Prazosin hydrochloride	Antihypertensive	Hydrogel	Extended drug release through transdermal route	[28]
Ciprofloxacin hydrochloride	Antibacterial	IPN scaffold	For quick and regulated wound healing	[29]
